# Recognising the role of radiographers in MR safety and the contributions of the European Federation of Radiographer Societies

**DOI:** 10.1186/s13244-024-01897-0

**Published:** 2025-01-17

**Authors:** Anke De Bock, Jonathan McNulty, Andrew England, ECSO-MRI Consortium, Anke De Bock, Jonathan McNulty, Andrew England

**Affiliations:** 1 Medical Imaging and Radiotherapy, Odisee University of Applied Sciences, Brussels, Belgium; 2 Radiography and Diagnostic Imaging Section, School of Medicine, University College Dublin, Dublin, Ireland; 3 Discipline of Medical Imaging and Radiation Therapy, School of Medicine, University College Cork, Cork, Ireland

Dear Editor,

We commend the timely publication ‘*The European MR Safety Landscape,*’ which underscores the critical need for harmonised MR safety practices across Europe [1]. However, it is important to address a notable omission: the article does not reference the extensive contributions from the European Federation of Radiographer Societies (EFRS), specifically our foundational work on MR safety standards and roles, including the ‘Magnetic Resonance Safety Officer (MRSO) Role Descriptor: An European Qualifications Framework (EQF) benchmarking document’ [2] or the current European Curriculum for Safety Officers in Magnetic Resonance Imaging (ECSO-MRI) project, co-funded by the Erasmus+ Programme of the European Union which involves five European universities and the EFRS who are working towards a safer MR environment through the development of a harmonised, university accredited, and European Credit Transfer System (ECTS) based European MRI safety curriculum for radiographers.

Radiographers are essential to MR safety, often serving as the primary guardians of patient and staff safety within the MR environment. The EFRS has led several initiatives to formalise and support this responsibility, most notably through the MRSO Role Descriptor [2], which provides a clear outline of the competencies, duties, and knowledge radiographers need to fulfil the MRSO role effectively. This document is a cornerstone of MR safety guidance, designed to ensure that radiographers are equipped to manage patient screening, emergency protocols, and MR safety regulations consistently across healthcare facilities, and is in keeping with the 2016 multi-society consensus guideline [3].

In addition to the MRSO Role Descriptor, the ECSO-MRI project is a collaborative initiative aimed at enhancing MR safety education and establishing a comprehensive curriculum for MR safety training of radiographers across Europe. The project’s goal is to ensure that radiographers have access to standardised, university-accredited training that addresses the latest safety protocols and technological advancements. By developing a unified educational framework, ECSO-MRI seeks to bridge existing gaps in MR safety training and support a more cohesive approach to MR safety across countries and institutions. In recognition of the advanced levels of MR safety knowledge, skills, and competence required for radiographers to fulfil the MRSO role, the advanced MR safety content, in the form of a standalone module/programme, will be delivered at and map to EQF Level 7 (Masters Level). The four-tier/module structure, with three parallel tracks in Module 3, described in the recent publication [1], aligns well with the approach being taken within the ECSO-MRI project, with the scope of the project being on the education and training needs of radiographers working in MRI rather than radiologists or medical physics experts. The curriculum, which is currently being piloted, was informed by a Europe-wide survey of educational institutions involved in the MR safety education and training of radiographers, at undergraduate and/or postgraduate levels, along with a survey of individual radiographers across Europe on their MR safety-related learning needs and preferences (both surveys will hopefully be published shortly).

The omission of these initiatives from the recent publication’s discussion represents a missed opportunity to highlight established resources and ongoing projects that directly address the need for consistency and high standards in MR safety. Both the MRSO Role Descriptor and the ECSO-MRI project align closely with the harmonisation and standardisation efforts that the article advocates, providing practical and effective solutions that have already been implemented across various European healthcare settings.

In conclusion, while this publication on the ‘*The European MR Safety Landscape*’ has made an excellent contribution to understanding the current MR safety landscape, it is essential to recognise that this landscape cannot be fully understood without considering the pivotal role of radiographers and complementary European activities. The EFRS’s contributions, including the MRSO Role Descriptor and the ECSO-MRI project, are fundamental to building a safe and standardised MR environment across Europe. The ongoing efforts by radiographers and the EFRS must be included in this dialogue, as they are integral to the continuous improvement and future development of MR safety standards.

Thank you for the opportunity to provide this perspective on the evolving landscape of MR safety.
